# A Recombinant *Novirhabdovirus* Presenting at the Surface the E Glycoprotein from West Nile Virus (WNV) Is Immunogenic and Provides Partial Protection against Lethal WNV Challenge in BALB/c Mice

**DOI:** 10.1371/journal.pone.0091766

**Published:** 2014-03-24

**Authors:** Angella Nzonza, Sylvie Lecollinet, Sophie Chat, Steeve Lowenski, Emilie Mérour, Stéphane Biacchesi, Michel Brémont

**Affiliations:** 1 UR0892 Unité de Virologie et Immunologie Moléculaires, INRA, CRJ, Jouy en Josas, France; 2 UMR 1161 Virologie, INRA, ANSES, UPEC ENVA, Maisons-Alfort, France; 3 UR1196 Unité Génomique et Physiologie de la Lactation, Plateau de Microscopie Électronique, INRA, CRJ, Jouy-en-Josas, France; Thomas Jefferson University, United States of America

## Abstract

West Nile Virus (WNV) is a zoonotic mosquito-transmitted flavivirus that can infect and cause disease in mammals including humans. Our study aimed at developing a WNV vectored vaccine based on a fish Novirhabdovirus, the Viral Hemorrhagic Septicemia virus (VHSV). VHSV replicates at temperatures lower than 20°C and is naturally inactivated at higher temperatures. A reverse genetics system has recently been developed in our laboratory for VHSV allowing the addition of genes in the viral genome and the recovery of the respective recombinant viruses (rVHSV). In this study, we have generated rVHSV vectors bearing the complete WNV envelope gene (E_WNV_) (rVHSV-E_WNV_) or fragments encoding E subdomains (either domain III alone or domain III fused to domain II) (rVHSV-DIII_WNV_ and rVHSV-DII-DIII_WNV_, respectively) in the VHSV genome between the N and P cistrons. With the objective to enhance the targeting of the E_WNV_ protein or E_WNV_-derived domains to the surface of VHSV virions, Novirhadovirus G-derived signal peptide and transmembrane domain (SP_G_ and TM_G_) were fused to E_WNV_ at its amino and carboxy termini, respectively. By Western-blot analysis, electron microscopy observations or inoculation experiments in mice, we demonstrated that both the E_WNV_ and the DIII_WNV_ could be expressed at the viral surface of rVHSV upon addition of SP_G_. Every constructs expressing E_WNV_ fused to SP_G_ protected 40 to 50% of BALB/cJ mice against WNV lethal challenge and specifically rVHSV-SP_G_E_WNV_ induced a neutralizing antibody response that correlated with protection. Surprisingly, rVHSV expressing E_WNV_-derived domain III or II and III were unable to protect mice against WNV challenge, although these domains were highly incorporated in the virion and expressed at the viral surface. In this study we demonstrated that a heterologous glycoprotein and non membrane-anchored protein, can be efficiently expressed at the surface of rVHSV making this approach attractive to develop new vaccines against various pathogens.

## Introduction

West Nile Virus (WNV) is a zoonotic arthropod-borne virus, belonging to the *Flaviviridae* family [1]. Birds are the main animal species infected by this virus, and are considered as the key WNV reservoirs [2]. Horses and humans can be incidentally infected by mosquito bites and are the most susceptible mammals to WNV infection. Accordingly, WNV epidemics have been recently reported in the USA causing severe neurological disorders in about 1% infected individuals, these latter forms being associated with fatal outcomes in about 10% of cases [3]. WNV genome consists of a positive single-stranded RNA molecule of about 11 Kilobases that is translated as a single polyprotein. WNV polyprotein is subsequently processed by cellular and viral proteases into the structural proteins C, M and E (E_WNV_) and 7 nonstructural (NS) proteins [1].

The E_WNV_ glycoprotein is the major surface and the most immunogenic protein in WNV virions [4]. Many studies on WNV vaccination have shown that recombinant E_WNV_ protein or DNA or viral vectors bearing the E_WNV_ gene can induce a strong and protective anti- E_WNV_ antibody and/or cellular response in various animal species [5]–[17]. Moreover a number of veterinarian vaccines against WNV have been developed in the past, based on DNA or gene vectors expressing WNV prM/E antigens. [18], [19]. The E_WNV_ glycoprotein is composed of three domains (DI, DII and DIII) that are connected by flexible hinge regions [20]; domain III contains the receptor-binding region [21] and most of the type-specific and potentially neutralizing B-cell epitopes [22], [23]. Domain III by itself has been shown to be sufficient to induce a protective immune response [6], [9], [11], [14].

Novirhabdoviruses like the Infectious Hematopoietic Necrosis Virus (IHNV) and the Viral Hemorrhagic Septicemia Virus (VHSV) infect trout, a fresh water fish species living at an optimal temperature ranging from 10 to 15°C. As a consequence, IHNV and VHSV have been adapted to grow in fish and in fish cell culture as well, at a comparable temperature range with a maximum temperature of 20°C and are naturally inactivated at higher temperatures [24]. As for mammalian rhabdoviruses, fish novirhabdoviruses are composed of a non-segmented negative-sense single-stranded RNA genome of about 11 kilobases which encodes five structural proteins, the nucleoprotein N, the polymerase-associated protein P, the matrix protein M, the unique surface glycoprotein G and the RNA-dependent RNA polymerase L. In addition, novirhabdoviruses encode for a nonstructural NV protein of unknown function. We have previously developed a reverse genetics system for both IHNV and VHSV novirhabdoviruses [25], [26] allowing the manipulation of the RNA viral genome such as the introduction of an additional cistron coding for an heterologous protein and the recovery of a recombinant virus (rVHSV) expressing the gene of interest [26], [27]. In the present study we show that VHSV can not only be used as a gene vector but also as an antigen-presenting platform for vaccination purposes. In the current study the glycoprotein E_WNV_ was chosen as a model antigen. Because VHSV buds from the plasma membrane, in areas where its G glycoprotein accumulates, while WNV is known to bud from the endoplasmic reticulum (ER) membranes where E_WNV_ is located, chimeric E_WNV_ constructs were realized to enhance its cell surface expression and to drive its incorporation into the VHSV virion envelope. The G signal peptide (SP_G_) from IHNV and the transmembrane domain (TM_G_) from VHSV were genetically fused to the amino and carboxy termini of the E_WNV_ protein and E_WNV_ domains. Here, we provide evidences that both the complete E_WNV_ protein and E_WNV_ domains can be inserted in the viral membrane of rVHSV and that they are able to induce a protective immunity, when serially administered to BALB/c mice, against a lethal WNV challenge.

## Materials and Methods

### Ethics statement

This study was performed in strict accordance with the French guidelines and recommendations on animal experimentation and welfare. All animal experiment procedures were approved by the local ethics committee on animal experimentation: ComEth Anses/ENVA/UPEC under permit number N° 13/12/11-3.

### Cells, virus and recombinant proteins

The recombinant VHSV (rVHSV) generated in this study were propagated in monolayer cultures of *Epithelioma papulosum cyprinid* (EPC) cells or bluegill fry-2 (BF-2) cells maintained at 14°C in Glasgow’s modified Eagle’s medium -HEPES 25 mM medium (GMEM-HEPES) supplemented with 2 mM L-glutamine and 2% of fetal bovine serum (FBS), as previously described [26]. Virus titers were determined by plaque assay on EPC cells under an agarose overlay (0.35% in Glasgow's modified Eagle's medium). At 4 days post-infection, cell monolayers were fixed with 10% formol and stained with crystal violet. Recombinant vaccinia virus expressing the T7 RNA polymerase, vTF7-3 [28], was kindly provided by B. Moss (National Institutes of Health, Bethesda, MD). The WNV strain used in this study is the Israël-98 strain [29] and was kindly provided by Philippe Desprès (Pasteur Institute, Paris, France). The recombinant E protein (rE_WNV_) and recombinant domain III of the E protein (rDIII_WNV_) from WNV used as positive controls in this study were also kindly provided by Philippe Desprès.

### Construction of rVHSV expressing the WNV E glycoprotein (E_WNV_)

**Figure 1:**
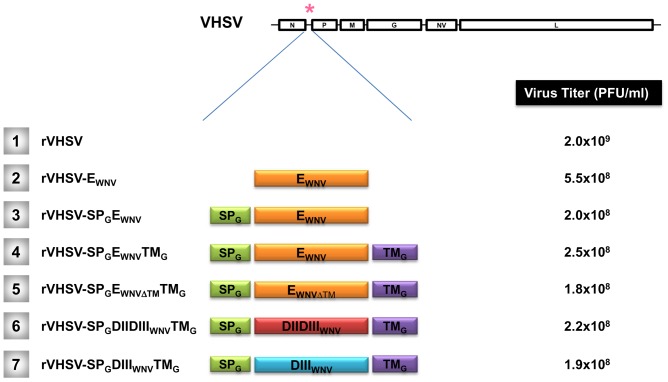
Recovery of six rVHSV expressing WNV antigens by reverse genetics. Six recombinant viruses containing an expression cassette between the N and P genes were recovered. These rVHSV expressing the entire WNV E glycoprotein (E_WNV_; #2), or E_WNV_ fused to the signal peptide (SP_G_) of the glycoprotein G of IHNV (SP_G_E_WNV_; #3), or E_WNV_ fused to the SP_G_ of the IHNV G and the transmembrane region (TM_G_) of the VHSV G (SP_G_E_WNV_TM_G_; #4), or fragments of E_WNV_ fused to SP_G_ and TM_G_: the ectodomain part of E_WNV_ (SP_G_E_WNVΔTM_TM_G_; #5), the domain III alone (SP_G_DIII_WNV_TM_G_; #7) or associated with a portion of domain II (SP_G_DIIDIII_WNV_TM_G_; #6). The titer of each rVHSV is indicated on the right.

The WNV E glycoprotein gene (1503 nucleotide in length) has been amplified by reverse transcription-polymerase chain reaction (RT-PCR) from the genomic viral RNA, extracted from the WNV Israël-98 strain using the QIAamp Viral RNA minikit (Qiagen) and the following primers 5′EWNV and 3′EWNV (**Table 1**). To generate a recombinant VHSV expressing the E glycoprotein from WNV, we used a pVHSV-GFP plasmid containing a full length copy of the VHSV cDNA genome and an expression cassette inserted between the N and P genes and composed by an additional cistron encoding the GFP (green fluorescent protein) flanked by the VHSV gene start and gene end signals. The GFP open reading frame (ORF) was removed from the additional cassette by *SpeI* and *SnaBI* restriction enzyme digestions. The *SpeI/SnaBI*-digested WNV-derived E PCR product was inserted into the *SpeI/SnaBI*-digested plasmid pVHSV-GFP, leading to pVHSV-E_WNV_. The rVHSV expressing the WNV E (rVHSV-E_WNV_; **Fig. 1** #2) was recovered by transfection of pVHSV-E_WNV_ together with the expression plasmids pT7-N, pT7-P and pT7-L derived from VHSV, in EPC cells infected with vTF7-3 vaccinia virus [28], as previously described (for a review see [30]). The supernatant of the infected and transfected cells were used to infect fresh EPC cells. After 2 passages, the titer was determined and the virus was stored at –80°C until use.

**Table 1:** Primers used in this study.

Primers	Sequence 5' to 3'	Restriction enzyme	Modified or added nucleotides
5′EWNV	GGATCC**ACTAGT** ATGTTTAACTGCCTTGGAATGAGCAACAG	Spe I	ATG
3′EWNV	GAATTC**TACGTA** TTAAGCGTGCACGTTCACGGAGAGGAAGAGCAG	SnaB I	TTA
SPIHH1	**ACTAGT** ATGGACACCACGATCACCACTCCGCTCATTCTCATTCTGATCACCTGCGGAGCAAACAGCA	Spe I	ATG
SPIHN2	**ACTAGT** TGCTGTTTGCTCCGCAGGTGATCAGAATGAGAATGAGCGGAGTGGTGATCGTGGTGTCCATA	Spe I	ATG
SPIHNDIIIF	**ACTAGT** ATGGACACCACGATCACCACTCCGCTCATTCTCATTCTGATCACCTGCGGAGCAGCTAGCGGAACAACCTATGGCGTCTGTTCAAAGG	Spe I, NheI	ATG
SPIHNF	**ACTAGT** ATGGACACCACGATCACCACTCCGC	Spe I	ATG
DIIITMVHSR	GGCCCCTCCCACAACCCCCATCCCAGATAACGCTCCTTTGAGGGTGGTTGTAAAGG		
DIIITMVHSF	CCTTTACAACCACCCTCAAAGGAGCGTTATCTGGGATGGGGGTTGTGGGAGGGGCC		
EPsiIF	CGGCACGA**TTATAA**CAAGACAAAC**AACTAG**TATGG	Psi I, Spe I	ATG
EPsiIR	GAGAAATTC**TTATAA**TCGTGCCGTTTTTTTCTATCTATG	Psi I	
MUTSTOP	CCGTGAACGTGCACGCT*GCA*TACGTACATAGATAGAAAAAAACGG		***TA***A
VHSTMF	**TACGTA** TTATCTGGGATGGGGGTTGTGG	SnaB I	
VHSTMR	**TACGTA** TCAGACCGTCTGACTTCTAGAGAACTGC	SnaB I	
MUTTMNRUI	CCACCCTCAAAGGA*CGTTT*ATCTGGGATGGGG	NruI	***TCGCG*** **A**
NheEF	**GCTAGC** TTTAACTGCCTTGGAATGAGCAACAGAGAC	Nhe I	
NruER	**TCGCGA** CCTATCACGATTGATGCCCATCC	NruI	
NHEDIIF	**GCTAGC** GGGATTGACACCAATGCATACTACGTGATG	Nhe I	
NRUDIIIR	**TCGCGA** CGCTCCTTTGAGGGTGGTTGTAAAGG	NruI	
WN3′NC-F10538	GAGTAGACGGTGCTGCCTGC		
WN3′NC-R10627	CGAGACGGTTCTGAGGGCTTAC		
ACTB-966F	CAGCACAATGAAGATCAAGATCATC		
ACTB-1096R	CGGACTCATCGTACTCCTGCTT		

Restriction enzyme sites are boldfaced; added ATG or TAA codons are underlined; mutated nucleotides are italicized.

### Construction of rVHSV expressing the chimeric proteins SP_G_E_WNV_, SP_G_E_WNV_TM_G_, SP_G_E_WNVΔTM_TM_G_, SP_G_DIIDIII_WNV_TM_G_ and SP_G_DIII_WNV_TM_G_

Five additional chimeric constructs were engineered pVHSV-SP_G_E_WNV_, pVHSV- SP_G_E_WNV_TM_G_, pVHSV-SP_G_E_WNVΔTM_TM_G_, pVHSV-SP_G_DIIDIII_WNV_TM_G_ and pVHSV-SP_G_DIII_WNV_TM_G_ (**Fig. 1**; recombinant viruses #3, 4, 5, 6 and 7 respectively). The signal peptide derived from the IHNV G glycoprotein sequence (SP_G_) was amplified by PCR using specific primers (**Table 1**; SPIHN1 and SPIHN2) and inserted in pVHSV-E_WNV_ upstream to the E_WNV_ gene after digestion with the restriction enzyme *SpeI*, leading to pVHSV-SP_G_E_WNV_ (**Fig. 1** #3). The pVHSV-SP_G_E_WNV_TM_G_ construct was generated using the two *PsiI* restriction sites present on both sides of the expression cassette. These restriction sites allowed removing the SP_G_E_WNV_ sequence from pVHSV-SP_G_E_WNV_. The fragment *PsiI* was amplified by PCR using the primers EPsiIF and EPsiIR (**Table 1**) and then was cloned into the universal plasmid pJET1.2 (ThermoScientific) according to manufacturer’s recommendations. The transmembrane region derived from the VHSV G glycoprotein sequence (TM_G_) was amplified using specific primers (VHSTMF and VHSTMR; **Table 1**) and inserted in the pJET1.2-SP_G_E_WNV_ after digestion by the *SnaBI* restriction enzyme. The stop codon at the end of the E gene was then changed to a neutral codon (GCA encoding an alanine) by site-directed mutagenesis using the MUTESTOP primer (QuikChange Multi Site-Directed Mutagenesis Kit; Stratagene). Finally, the SP_G_E_WNV_TM_G_ fragment was removed from the pJET1.2 vector by digestion with the *PsiI* restriction enzyme and cloned into the pVHSV cassette previously digested with *PsiI*, leading to pVHSV-SP_G_E_WNV_TM_G_ construct (**Fig. 1**; #4).

The sequence encoding the domain III of WNV E glycoprotein (amino acid 300 to 416) was fused at the 5′ and 3′ends to the SP_G_ and TM_G_ sequences, respectively. For that, three successive PCR, including a fusion PCR, were conducted as depicted in **Figure S1**. Nucleotide sequences of the primers used for the PCR reactions are indicated in the **Table 1**. The final domain III ORF flanked by the SP and TM domain of G was first cloned in the pJET1.2 vector (pJET1.2-SP_G_DIII_WNV_TM_G_) and then was inserted in the expression cassette of pVHSV using the *SpeI* and *SnaBI* restriction sites, leading to pVHSV-SP_G_DIIITM_G_ construct (**Fig. 1**; #7). The sequences encoding the WNV E glycoprotein ectodomain (E_WNVΔTM_) and the domains II and III (DIIDIII_WNV_) were amplified by PCR from the cDNA encoding the complete E glycoprotein using specific primers (**Table 1**). A *NruI* restriction site was introduced by directed mutagenesis at the end of the coding sequence of the DIII_WNV_ in the pJET1.2-SP_G_DIII_WNV_TM_G_ (**Fig. S1**). A unique *NheI* site at the beginning of the DIII sequence was formed when the SP_G_ sequence was fused to the DIII sequence. After removing the DIII from this plasmid, the E_WNVΔTM_ and DIIDIII_WNV_ fragments were cloned between the SP_G_ and TM_G_ sequences using the restriction sites *NheI* and *NruI*. The final ORF were inserted in the expression cassette of pVHSV, leading to pVHSV-SP_G_E_WNVΔTM_TM_G_ and pVHSV-SP_G_DIIDIII_WNV_TM_G_ constructions, respectively (**Fig. 1**; #5 and 6). The sequence of the additional expression cassette of each modified pVHSV was confirmed by nucleotide sequencing. All the recombinant viruses were rescued and amplified as described above. After 2 passages on EPC cells, the titer was determined and the viruses were stored at –80°C until use.

### Virus production and purification

Recombinant viruses were mass produced, clarified by low-speed centrifugation (4,000 rpm for 15 min) and were purified by ultracentrifugation at 36,000 rpm in a SW41 Beckman rotor for 4 hours through a 25% (w/v) sucrose cushion in TEN buffer (10 mM Tris-HCl (pH∶7.5), 150 mM NaCl, 1 mM EDTA (pH 8)). Pellet was resuspended in TEN buffer and aliquots were analyzed on a sodium dodecyl sulfate (SDS)-12% polyacrylamide gel stained with Coomassie blue and also examined by electron microscopy observation (Zeiss EM902 85 Kvolt) to estimate the purity of the virus preparations. The viral protein yield (in micrograms) of each preparation was quantified by using the Micro BCA assay protein quantification Kit (Pierce) in accordance with the manufacturer’s instructions.

### Indirect immunofluorescence analysis on fixed and living cells

BF-2 cells grown in 24-well plates were infected with the rVHSV (passage 2, MOI of 2). At 48h post-infection, cells were fixed with a mixture of ethanol and acetone (1∶1, vv) at –20°C for 20 min and washed with PBS. Primary mouse monoclonal antibodies against E_WNV_ (mAb8150 diluted 1∶100; Chemicon International), E24 [31], [32] provided by Dr Philippe Desprès (dilution 1∶1500) were incubated in PBS-Tween 0.05% for 45 min at room temperature (RT) and washed 3 times with PBS-Tween 0.05%. Cells were then incubated with Alexa Fluor 488-conjugated anti-mouse immunoglobulins diluted to 1∶3,000 (Invitrogen) in PBS-Tween 0.05% for 45 min at RT. Cell monolayers were then visualized with a UV-light microscope (Carl Zeiss).

For live cells, infected cell monolayers were directly incubated with primary mouse antibodies in GMEM 10% FBS culture medium for 45 min at RT. After 3 washes with the same medium, cells were incubated with Alexa Fluor 594-conjugated anti-mouse immunoglobulins (dilution 1:3,000) for 45 min at RT. Three washes were performed and cell monolayers were then visualized with a UV-light microscope (Carl Zeiss).

### SDS-polyacrylamide gel electrophoresis and Western blot assay

Aliquots of sucrose-purified recombinant viruses or recombinant WNV E protein were separated on a sodium dodecyl sulfate (SDS)-12% polyacrylamide gel and either stained with Coomassie blue or electrotransferred onto a nitrocellulose membrane (ECL Amersham Hybond membrane; GE Healthcare) using a semidry electroblotting system (Biorad). The membrane was saturated in Tris Buffer Saline containing 0.05% of Tween20 (TBST) supplemented with 5% of milk for 1h at RT, then incubated with mouse primary antibody against E or sera from immunized mice in TBST (dilution 1:33) for 1h at RT. After three washes with TBST, the membrane was incubated for 1h at RT with horseradish peroxidase-conjugated anti-mouse antibody (1∶5,000; P.A.R.I.S.) in TBST. After washing with TBST, peroxidase activity was revealed by incubation with ECL Western Blotting Detection Reagents (GE Healthcare) according to the manufacturer’s instructions.

### Immunogold electron microscopy

Formvar-coated EM grids (300 meshs) were turn over a drop (40 μl) of sucrose purified recombinant viruses for 5 min. After virus adsorption, the grids were washed with PBS for 3 min and then fixed with 1% paraformaldehyde (PAF) for 5 min. Excess of PAF was removed by incubating the grids in PBS for 3 min. Grids were saturated twice with PBS containing 1% of bovine serum albumin (BSA) and 0.1% of complemented BSA (cBSA (Tebu)) for 15 min and then incubated with mouse anti-WNV E or anti-VHSV G primary antibodies in PBS-1% BSA-0.1% cBSA for 2 h. After 4 washes, the grids were incubated for 1 h with an anti-mouse antibody coupled with gold particles (5 nm in diameter; dilution 1∶50 (British Biocell International – TEBU, France) in PBS-1% BSA-0.1% cBSA. The grids were washed with PBS 4 times for 3 min, fixed with 2.5% of glutaraldehyde in PBS for 5 min and contrasted with 1% of aluminium molybdate for 20–30 s. All these steps were performed at RT. The grids were observed using a transmission electron microscope (Zeiss EM902) operated at 80 kV. Microphotographies were acquired with a charge-coupled device camera MegaView III CCD camera and analysed with ITEM Software (Eloïse, France) MIMA2 Platform UR1196, INRA-CRJ (www6.jouy.inra.fr/mima2).

### Quantitative RT-PCR

Total RNA was extracted from blood of WNV-challenged mice by using the QIAamp Viral RNA purification Kit (Qiagen), according to the manufacturer’s instructions. Specific WNV RNA was reverse-transcribed and amplified following the conditions described by [33] and using the AgPath-ID One-Step RT-PCR Kit (Applied Biosystems), with primers WN3′NC-F 10538 and WN3′NC-R 10627 located in the 3′ untranslated region (UTR) and primers ACTB-966F and ACTB-1096R for amplification of cellular β-actin mRNA (Table 1). Primers were used at 0.4 μM and probes (WN3′NC-probe 10564c (5′-FAM-ACCCAGTCCTCCTGGGGT-MGB-3′) and ACTB1042-67 (5′-VIC-TCGCTGTCCACCTTCCAGCAGATGT-TAMRA-3′) at 0.2 μM. Reaction mixtures (25 μl) contained 5 μl of RNA extracted from each sample. Amplification was performed in an AB 7300 Real-Time PCR system (Applied Biosystems). The thermal profiles of the reaction were as follows: 45°C for 10 min (RT), 95°C for 10 min (Taq activation), and 40 cycles at 95°C for 15 s and 60°C for 1 min (amplification). After checking the amount of actin, similar in each sample, the genomic RNA of WNV was quantified as the number of copies per ml of blood collected and mice were considered positive for viremia when number of RNA copies/ml reached 100 (detection threshold).

### Immunization experiments in BALB/c mice

In this first experiment, five BALB/c mice (6-week-old) were purchased from Janvier (Le Genest-Saint-Isle, France) and were let to acclimate in a BSL3 facility for one week before starting the experiment. Mice were subcutaneously immunized with 10 μg of rVHSV-SP_G_E_WNV_ in the presence of Freund's adjuvant (Sigma) (complete Freund’s adjuvant for the first immunization and the incomplete Freund’s adjuvant for the two last immunizations (ratio antigen/adjuvant (v/v) equal to 1:2). Mouse blood was harvested at the mandibular vein before each inoculation. Sera were collected from blood the next day, after a night at 4°C and a centrifugation step at 4°C for 10 min at 3,000 rpm and were stored at –20°C.

### WNV challenge in vaccinated mice

**Figure 2:**
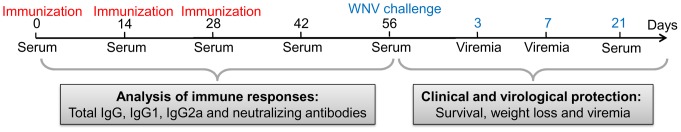
Schematic diagram of immunization and challenge schedule. 5-week old BALB/c mice were immunized three times with different antigens at two-week interval. Serum samples from immunized mice were collected every two weeks in order to assess antibody production. One month after the last immunization, the mice were challenged intraperitoneally with a lethal dose (1,000 PFU) of WNV (Israël-98 strain). Blood samples were collected on days 3 and 7 post-challenge to quantify the viremia by qRT-PCR. Mice were sacrificed on day 21 post-challenge, after a last serum sample was harvested.

Groups of 5-week-old female BALB/c mice (n = 12/group) were subcutaneously immunized three times with purified recombinant viruses (10 μg per mouse for each injection) in the presence of Freund's adjuvant, as described above. The three immunizations were given at two-week intervals (**Figure 2**). The positive control groups were immunized three times with rE_WNV_ or rDIII_WNV_ (1 μg per mouse for each injection) in the presence of Freund's adjuvant. The negative control group was injected three times with TEN buffer or empty rVHSV. Sera from each mouse were collected every two weeks up to the WNV challenge on day 56. Twenty-eight days after the last immunization, mice were challenged intraperitoneally with a lethal dose (1,000 PFU) of WNV (Israël-98 strain). On days 3 post-infection, blood samples were collected in EDTA and viral RNA was quantified by quantitative RT-PCR (as described above). Surviving mice were bled and sacrificed on day 21 post-challenge and WNV infection was confirmed by analyzing mice seroconversion (commercial competition ELISA, see below).

Humane endpoints were used during the survival study. The criteria used to define the ending point were the followings: every animal experiencing at least two of the listed clinical signs, e.g weight loss superior to 10%, anorexia, ataxia, loss of balance or paresia was humanely euthanized. Condition of the animals was monitored twice a day from day 6 to day 14 post-infection, corresponding to the time period during which clinical signs were reported. The animals were euthanized by cervical dislocation.

No systemic treatment was used, apart from anaesthetics, provided that they could modify the course of infection. Local treatments were accepted but not needed. Intraperitoneal challenge with West Nile virus was performed under general anaesthesia with a mixture of ketamine (100 mg/kg) and xylazine (10 mg/kg).

### ELISA Test

Measurement of total IgG, IgG1 and IgG2a in pooled sera.

The titer and the isotype profile of antibodies directed against E_WNV_ glycoprotein in the serum of immunized mice were evaluated by indirect ELISAs. One hundred nanograms of rE_WNV_ diluted in PBS were adsorbed per well on a 96-well plate (Immuno Plate Maxisorp Surface; Nunc) overnight at 4°C. After five washes with PBS-Tween 0.1%, each well was saturated with 100 µl of PBS-Tween 0.1% containing 3% of milk for 1 h at 37°C. The plates were then incubated for 1 h at 37°C with serial two-fold dilutions of pooled sera from each group of mice in PBS-Tween 0.1% containing 1% of milk. The lowest dilution tested was 1∶50 and the higher was 1∶204,800. Following a second washing step with PBS-Tween 0.1%, the plates were incubated for 1 h at 37°C with 100 μl of different peroxidase-conjugate secondary antibodies: a rabbit antibody directed against mouse total IgG diluted to 1∶5,000 (Jackson), a rabbit antibody against mouse IgG1 diluted to 1∶5,000 (Molecular Probes) and a rabbit antibody against mouse IgG2a diluted to 1∶5,000 (Invitrogen). Finally, the plates were washed five times with PBS-Tween 0.1% and specific antibodies were detected by the addition of TMB substrate (3, 3′, 5, 5′-tetramethylbenzidine; KPL). The reaction was stopped by the addition of phosphoric acid (H_3_PO_4_). The optical density (OD) was measured at 450 nm using an ELISA plate reader (Multiskan EX, Thermolab system) and the Ascent 2.6 software. The antibody titer is the reciprocal of the last dilution for which OD value was at least two-fold higher than the OD value of the pre-immune controls (sera taken before the first immunization).

#### Analysis of the individual IgG production

Individual production of total IgG was also analyzed by performing indirect IgG ELISA tests. Sera were collected from each mouse on day 56 when antibody production reached its plateau. This test was performed in duplicate for each serum at a single dilution of 1/100 and following the protocol described above. OD values for each individual are directly represented on the charts after removal of blank OD (OD measured in absence of serum).

#### Competition ELISA test

The "ID- Screen West Nile Competition" test (ID-Vet) is a commercial test that detects immunoglobulins against WNV prM/E in sera from virtually every animal species. This solid-phase blocking ELISA was performed and interpreted according to manufacturer’s instructions.

### Micro-neutralization Assays

Sera collected from immunized mice were heat inactivated at 56°C for 30 min. An aliquot of each serum was three-fold diluted (from 1∶10 to 1:7,290) in a final volume of 50 μl MEM. Each serum dilution was incubated with 50 μl MEM containing 100 TCID_50_ of Israël-98 strain at 37°C for 90 min in a tissue culture 96-well plate (Life Technologies). Subsequently, 2×10^4^ Vero cells in 100 μl of MEM containing 5% FBS, 1% pyruvate, 1% penicillin/streptomycin were added in each well and the plate was incubated for 72 h at 37°C in a CO2 incubator. The appearance of cytopathic effects was observed under a light microscope. Antibody titer was defined as the reciprocal of the last dilution at which cells were protected and a serum was considered positive if its titer exceeded 30.

## Results

### Characterization of the rVHSV expressing the WNV E and E-truncated forms

A series of rVHSV expressing various forms derived from the glycoprotein E of WNV have been generated as depicted in **Figure 1**. An expression cassette, containing the VHSV gene start and gene stop signals recognized by the viral polymerase and allowing the efficient expression of heterologous genes, has been introduced in the VHSV genome between the VHSV N and P cistrons, as previously described [26]. In most of the constructs encoding the chimeric genome rVHSV, the E gene (residues 1 to 501) or parts of the E (DIII residues 300–416; DII+DIII residues 182–416; and E_ΔTM_ residues 1–487) have been fused to a signal peptide (SP_G_) derived from the glycoprotein G of IHNV and a transmembrane domain (TM_G_) derived from the glycoprotein G of VHSV. A total of six rVHSV genomes were constructed as presented in Figure 1. Strategy to obtain the different constructs is detailed in Materials and Methods. All rVHSV were readily recovered through transfection of vTF7-3-infected EPC cells with pT7-N, pT7-P, pT7-L and the various pVHSV plasmid constructs, as previously described (for a review see [30]). Each rVHSV has been amplified through 2 passages in cell culture and titered. Each rVHSV reached high titers, ranging from 1.8×10^8^ to 2×10^9^ pfu/ml, as indicated in **Figure 1**. The increase in genome size of about 10% has little effect on viral replication.

### VHSV is able to express WNV derived antigens in fish cells

**Figure 3:**
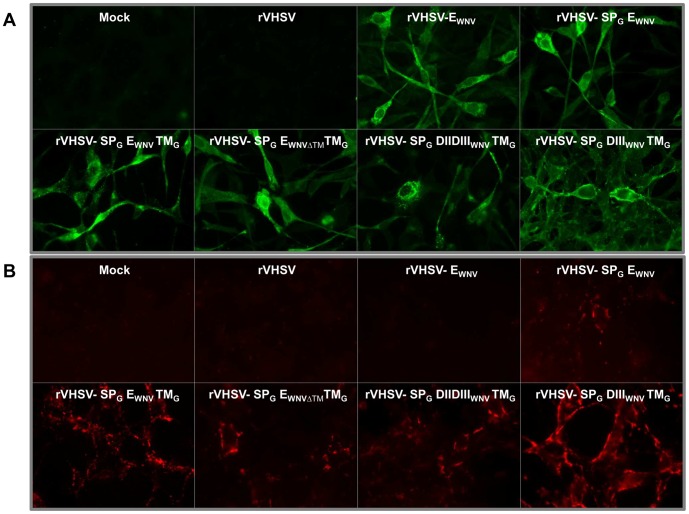
Expression of E_WNV_ antigens in rVHSV-infected cells. The expression of E_WNV_ antigens was assessed by indirect-immunofluorescence in BF-2 cells. The cells were infected or not infected (Mock) either with the empty vector (rVHSV) or the six recombinant viruses expressing E_WNV_ domains (as indicated). Cells were incubated for 48 h at 14°C. (A) At 48 h post-infection, cells were fixed and permeabilized with a mixture of alcohol/acetone, and protein expression was detected using a monoclonal antibody against E_WNV_ DIII (E24). (B) Detection of membrane expression of E_WNV_ antigens was performed on live cells using the E24 antibody, except for rVHSV-SP_G_DIIDIII_WNV_TM_G_ infected cells where E_WNV_ expression was achieved with mAb8150 (magnification X63).

The ability of each rVHSV to express the E_WNV_ or part of the E_WNV_ was first evaluated by an indirect immunofluorescence assay performed on infected and fixed BF-2 cells and using an anti-E_WNV_ monoclonal antibody. As shown in **Figure 3A**, all rVHSV expressed as expected the E_WNV_ protein or E_WNV_-derived domains in the cytoplasm of infected BF-2 cells, demonstrating the functionality of the additional insertion cassette in the VHSV genome. As the goal was to generate rVHSV presenting the antigen of interest at the viral surface, the expression of the E_WNV_ domains at the membrane of rVHSV-infected cells was analyzed. Immunofluorescence assays on living rVHSV-infected cells were performed. As shown in **Figure 3B**, with the exception of rVHSV-E_WNV_ infected cells_,_ all the cells infected with the various rVHSVs expressed the E_WNV_ antigens at the cell surface. The level of expression varied with the rVHSV considered, and the cells expressing the highest amount of E_WNV_ antigens at the cell membrane appeared to be the ones infected with the rVHSV-SP_G_DIII_WNV_TM_G_. Interestingly, entire E_WNV_ without the addition of the G signal peptide (SP_G_) was unable to reach the cell membrane and probably stayed entrapped in the ER as during WNV natural budding (figure 3B; rVHSV-E_WNV_). The addition of the SP_G_ and the TM_G_ facilitated the addressing and anchor of E_WNV_ protein and domains at the cell surface of the rVHSV-SP_G_DIII_WNV_TM_G_ and rVHSV-SP_G_E_WNV_TM_G_ infected cells. Note that plasma membrane expression of E_WNV_ antigen by rVHSV-SP_G_E_WNV_ and rVHSV-SP_G_E_WNVΔTM_TM_G_ infected cells was similar suggesting that the signal peptide was sufficient to promote the plasma membrane targeting. Our observations also suggested that the removal of the transmembrane region of E_WNV_, despite the addition of TM_G_ did not enhance its addressing to the plasma membrane.

### E_WNV_ domains are incorporated at the surface of the purified recombinant viruses

**Figure 4:**
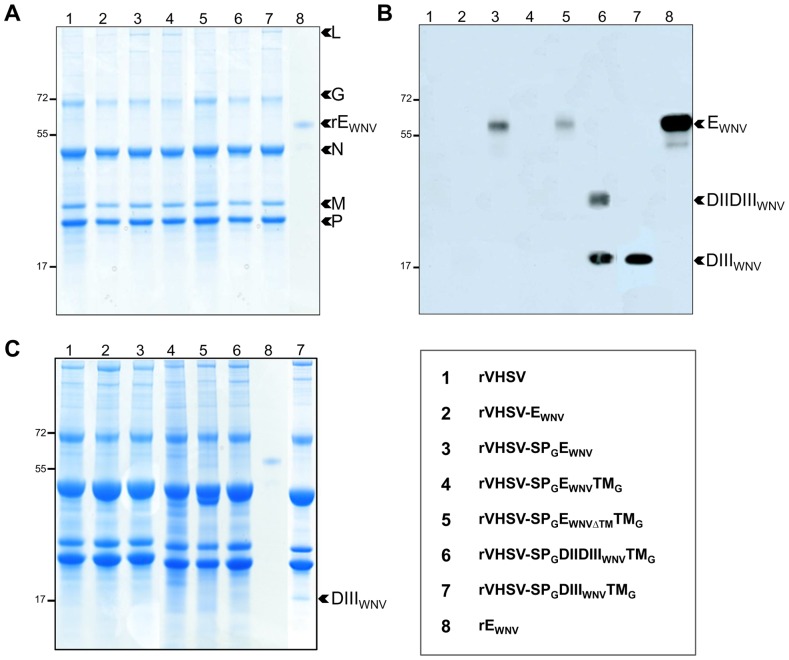
Analysis of virion incorporation of E_WNV_ antigens. Sucrose gradient-purified viral proteins were separated on a SDS-12% polyacrylamide gel. (A) Ten μg of total viral proteins were visualized after Coomassie blue staining. (B) Four μg (lanes 1 to 6) and 2 μg (lane 7) of total viral proteins were loaded. The gel was electrotransferred onto a nitrocellulose membrane and incubated with a mixture of mAb8150 and E24 anti-E_WNV_ antibodies. (C) Thirty μg of total viral proteins were visualized after Coomassie blue staining. Lane 8 corresponds to rE_VWN_ (1 μg was loaded on each gel).

Since VHSV buds from the plasma membrane, upon efficient addressing of WNV domains at the membrane of rVHSV-infected cells, it can be speculated that E_WNV_ antigens can be incorporated into the virion envelope. Consequently, next step was to investigate whether the E_WNV_ domains were incorporated in the rVHSV particles. All rVHSV viruses were purified on sucrose cushion, viral proteins were separated on SDS-PAGE and Western blot assays were achieved (**Figure 4**). The pellets of virus recovered after purification consisted mainly of five viral structural proteins (N, P, M, G and L), which were clearly distinguished in gel after migration and staining with Coomassie Blue (**Figure 4A**). No difference was observed between the chimeric constructs and empty rVSHV (lane 1). The presence of an expression cassette had no effect on total amount of incorporated viral proteins. E_WNV_ proteins were not visible after migration and staining of 10 micrograms of total proteins (lanes 2–7), while the migration of rE_WNV_ generated a band at about 55 kDa (as expected for the secreted portion of E_WNV_). **Figure 4B** shows that, with the exception of the rVHSV-E_WNV_, all the rVHSV have incorporated E_WNV_ antigens, and E_WNV_ domains were more efficiently incorporated that the entire E_WNV_. The absence of E_WNV_ in the rVHSV-E_WNV_ particles confirmed that E_WNV_ was not appropriately addressed at the plasma membrane in rVHSV-E_WNV_ infected cells (see immunofluorescence on living rVHSV-E_WNV_-infected cells; figure 3B). Surprisingly, no signal was detected in the purified rVHSV-SP_G_E_WNV_TM_G_ (lane 4) but subsequent assays demonstrated the presence of E_WNV_ in purified rVHSV-SP_G_E_WNV_TM_G_, at lower quantities than speculated with IFA. Indirect ELISAs with decreasing amounts of purified rVHSV-SP_G_E_WNV_TM_G_ particles and anti-E_WNV_ monoclonal antibody indicated that E_WNV_ expression in rVHSV-SP_G_E_WNV_TM_G_ was comparable to that detected in purified rVHSV-SP_G_E_WNV_ (data not shown). As already observed on nitrocellulose membranes stained with anti-E_WNV_ mAb, DIII_WNV_ was very abundant in purified rVHSV-SP_G_DIII_WNV_TM_G_ particles, and was already visible on a Coomassie blue-stained SDS-PAGE performed with 30 μg of total proteins (lane 7; **Figure 4C**). To be noticed that the western-blot analysis on rVHSV-SP_G_DIIDIII_WNV_TM_G_ revealed two bands corresponding to the molecular weight of DII-DIII and DIII. It is not yet clear whether this is due to a specific protein processing or due to an internal translation initiation.

**Figure 5:**
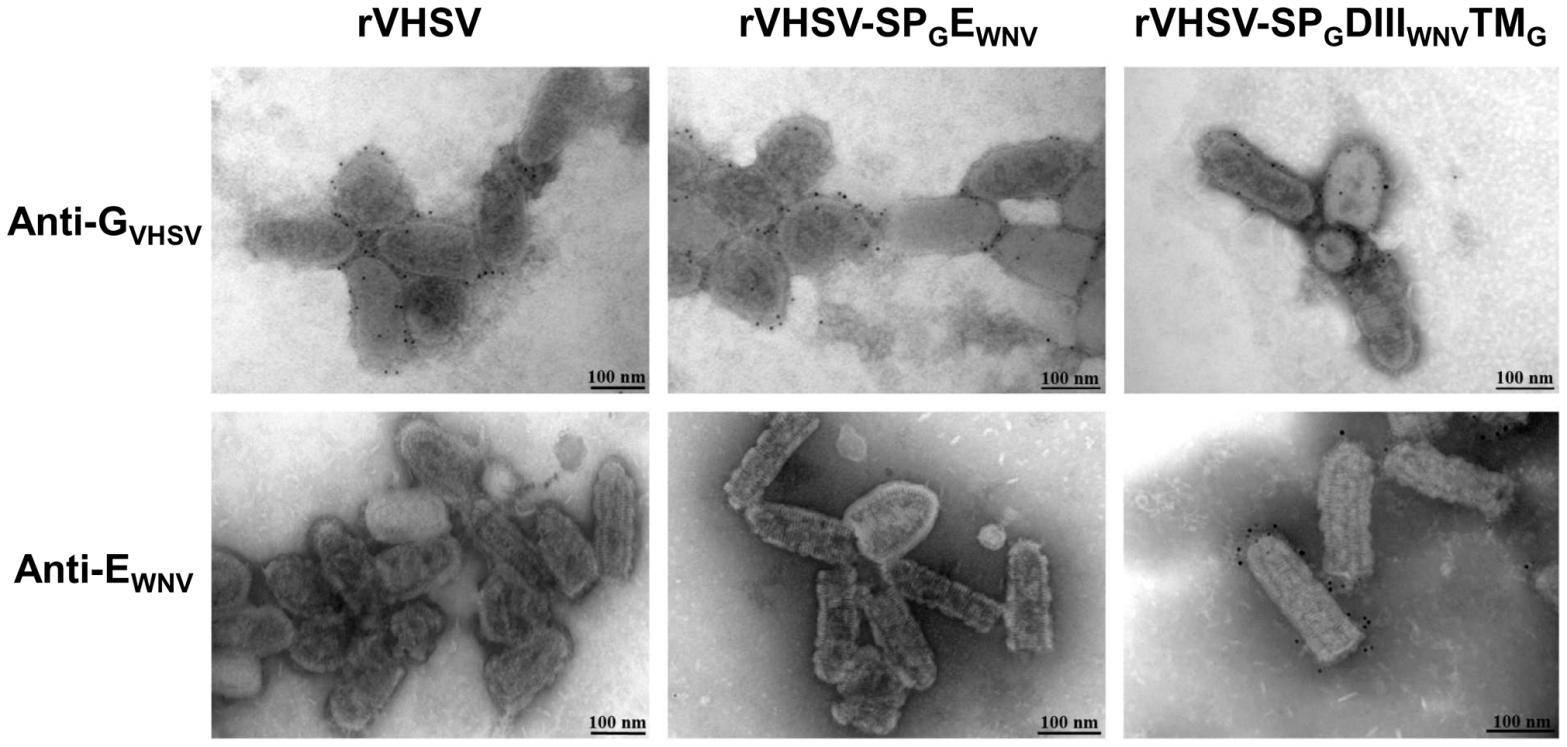
Detection of DIII_WNV_ at the virus surface of rVHSV-SP_G_E_WNV_ and rVHSV-SP_G_DIII_WNV_TM_G_ by immunogold. Sucrose purified-recombinant viral particles were adsorbed on electron microscopy nickel grids. After fixation, DIII_WNV_ and the glycoprotein G of VHSV (G_VHSV_) were detected using specific mouse primary monoclonal antibodies. These mouse primary antibodies were detected by an anti-mouse secondary antibody coupled with a gold particle (black dots of 5 nm in diameter). After negative staining, recombinant viral particles were observed by transmission electron microscopy.

To further specify where E_WNV_ antigens had been incorporated in rVHSV particles, purified viruses were observed by electron microscopy after immunogold labeling with either anti- E_WNV_ mAb (E24) or C10 mAb directed against the VHSV G as control. Recombinant viral particles had a typical morphology of rhabdoviruses, similar to that of rVHSV. The addition of E_WNV_ foreign sequences did not cause significant morphological changes. **Figure 5** illustrates the E_WNV_ and G labeling of rVHSV control and rVHSVs expressing the proteins SP_G_E_WNV_ and SP_G_DIII_WNV_TM_G_. In all three cases the VHSV G glycoprotein was detected at comparable levels. No signal was observed when empty rVHSV was incubated with anti-E_WNV_ antibody. Patchy staining was obtained with rVHSV-SP_G_E_WNV_ (**Figure 5**) and rVHSV- SP_G_E_WNV_TM_G_, SP_G_E_WNVΔTM_TM_G_ and SP_G_DIIDIII_WNV_TM_G_ reflecting the low incorporation of these proteins (data not shown). In contrast, DIII_WNV_ was highly expressed at the viral membrane (**Figure 5**), at a level apparently comparable to VHSV glycoprotein G.

### Antibody response in mice immunized with rVHSV-SP_G_E_WNV_

**Figure 6:**
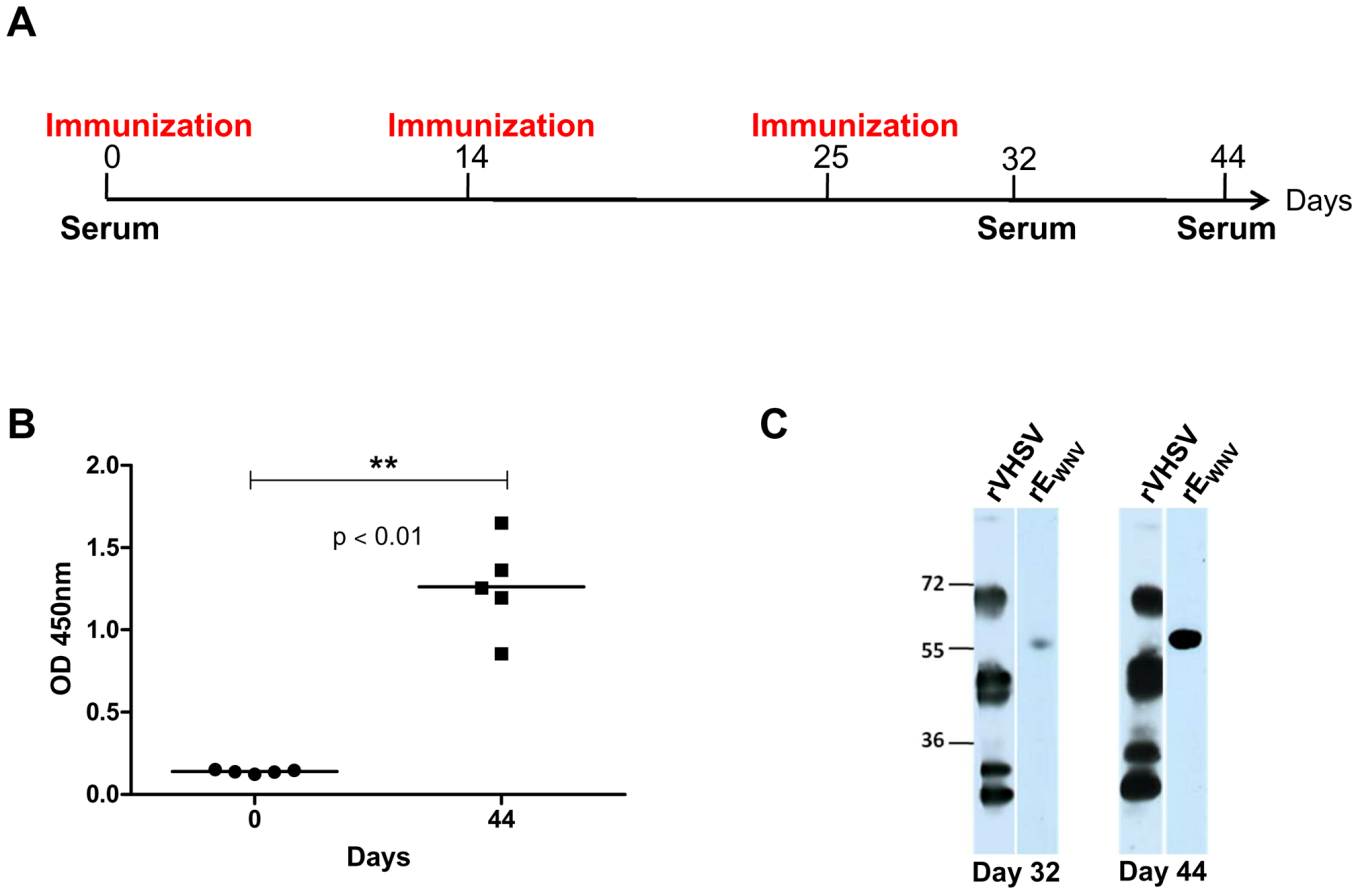
Purified rVHSV-SP_G_E_WNV_ is immunogenic and induces the production of antibodies against E_WNV_ in BALB/c mice. (A) Schematic diagram of immunization. Five 6-week-old BALB/c mice were injected subcutaneously with 10 μg of purified rVHSV-SP_G_E_WNV_ three times at two-week interval. Mice sera were taken before the first immunization (day 0), one week (day 32) and three weeks (day 44) after the last immunization. (B) The presence of antibodies against WNV E glycoprotein in the serum of immunized mice was analyzed by ELISA on days 0 and 44 after the last immunization. Statistical significance was determined by *t test*. (C) Example of antibody specificity against WNV E and VHSV structural proteins tested by Western Blotting. Two μg of total viral proteins from sucrose purified-VHSV and 1 μg of rE_WNV_ were separated on a SDS-12% polyacrylamide gel and electrotransferred in a nitrocellulose membrane. The mouse sera, harvested 1 and 3 weeks after the last immunization (day 32 and 44, respectively), were used as primary antibodies to detect the five structural proteins of VHSV (left lane) and the rE_WNV_ (right lane).

To examine whether rVHSV-SP_G_E_WNV_ is able to induce an antibody response in immunized mice, five 5-week old BALB/c mice were injected three times at day 0, 14 and 25 (**Figure 6A**) with 10 μg of rVHSV-SP_G_E_WNV_. At day 0, 32 and 44 post-inoculation, sera from each mouse were collected and anti-E_WNV_ immune response was evaluated by IgG indirect ELISA and Western blot assays. As shown in **Figure 6B**, the 5 immunized mice were positive in E_WNV_ IgG ELISA at day 44. The 5 inoculated mice showed OD values between 1 and 1.7, higher than those measured with pre-immune sera (less than 0.2). The observed difference in anti-E_WNV_ IgG before and after three inoculations was statistically significant. Presence of specific antibodies in the sera of one inoculated mouse was further confirmed by Western blot, with sera taken 1 and 3 weeks after the third inoculation (day 32 and 44, respectively) against the rVHSV on the one hand and against the rE_WNV_ on the other hand_._(**Figure 6C**). Although very few amounts of E_WNV_ was expressed at the surface of rVHSV particles, all the 5 immunized mice seroconverted against E_WNV_, and antibody production increased over time (with a higher production 3 weeks than 1 week after the third inoculation) (**Figure 6C**). Moreover, VHSV was highly immunogenic as shown in this figure; antibodies against four VSHV structural proteins (N, P, M and G) were present in the mouse serum on days 32 and 44. We demonstrated that VHSV was highly immunogenic in mice and thus favored the induction of an immune response against the heterologous antigen expressed by the rVHSV.

### Serial immunizations with rVSHV expressing entire E_WNV_ induce a Th2-oriented immune response and the production of neutralizing antibodies

All the recombinant rVHSV expressing E_WNV_ antigens were used to immunize mice in an attempt to correlate the induction of anti- E_WNV_ antibodies and E_WNV_ expression at the surface of rVHSVs and to select the recombinant virus inducing the most promising immune response. As depicted in **Figure 2**, the inoculation scheme included 3 injections every 2 weeks with 10 μg of each rVHSVs, as established during the first immunization assay.

Like during the first immunization trial (**Figure 6**), IgG production could be easily evidenced by indirect IgG ELISA in mice vaccinated with rVSHV-SP_G_E_WNV_, rVHSV-SP_G_E_WNVΔTM_TM_G_ (**Table 2**) or rVHSV-SP_G_E_WNV_TM_G_ (data not shown).

**Table 2:** rVHSV expressing WNV E antigens induce IgG and neutralizing antibody production.

Antigens			Immunogenicity			
	Dose	Number and route of immunization^a^	Total IgG^b^	IgG1^b^	IgG2a^b^	Number of animals with Nab against WNV^c^
TEN 1X	50 μl	3, SC	-	-	-	0/12
rE_WNV_	1 μg	3, SC	6,400	100,000	100,000	10/12
rDIII_WNV_	1 μg	3, SC	6,400	200,000	50,000	6/12
rVHSV	10 μg	3, SC	-	-	-	0/12
rVHSV-SP_G_E_WNV_	10 μg	3, SC	400	3,200	-	4/12
rVHSV-SP_G_E_WNVΔTM_ TM_G_	10 μg	3, SC	100	1,600	-	0/12
rVHSV-SP_G_DIIDIII_WNV_TM_G_	10 μg	3, SC	-	-	-	1/12

(a) 5-week-old BALB/c mice in groups of 12 animals were immunized three times by subcutaneous (SC) injection.

(b) The titers of total IgG, IgG1 or IgG2a produced in immunized mice were determined by ELISA. The sera of 12 mice per group were harvested the day of the challenge (day 56) and pooled. The titer is the reciprocal of the last dilution at which the OD value measured is two times higher than the negative control (serum from day 0 for each group).

(c) The production of neutralizing antibodies was demonstrated by WNV neutralization assay. The titer of neutralizing antibodies is the reciprocal of the last dilution for which no CPE could be observed. Individuals with serum neutralization titers ≥ 30 are considered as positive.

With the objective to determine the orientation of the immune response induced after immunization with rVHSVs, we sought to measure IgG1 and IgG2a production. A cellular-oriented immune response (T helper 1) is associated with a preponderant IgG2a response while a T helper 2 response (antibody-based) is associated with the production of IgG1 antibodies. Two groups of mice immunized with the rE_WNV_ and rDIII_WNV_ produced in insect cells were used as positive controls and showed the highest production of total IgGs, as well as isotypes IgG2a and IgG1, suggesting that these recombinant proteins induced mixed Th1 and Th2 immune responses. No IgG production was observed in the negative control groups (inoculated with rVSHV or the inoculation buffer) (**Table 2**). Mice immunized with rVHSV-SP_G_E_WNV_, or rVHSV-SP_G_E_WNVΔTM_TM_G_ only produced IgG1 antibodies (**Table 2**), suggestive of a Th2-biased response. Surprisingly, the rVHSV expressing E_WNV_ domains II-III (**Table 2**) or domain III alone (data not shown) were unable to induce any IgG production

**Figure 7:**
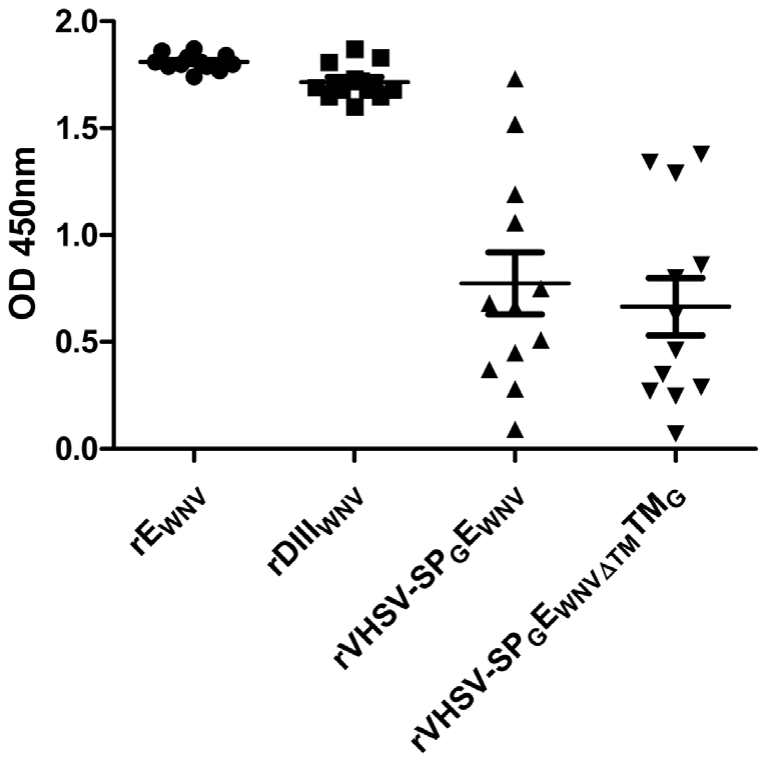
Individual production of total IgG after immunization with rVHSV-SP_G_E_VWN_ and rVHSV-SP_G_E_VWNΔTM_TM_G_. 5-week-old BALB/c mice in groups of 12 individuals were immunized three times with different antigens as indicated (see fig.2 for schedule details). Antibody production by each mouse was assessed by ELISA with individual sera collected on day 56 just before the WNV challenge. Each serum was diluted to 1∶100. OD values for each individual are represented directly after removal of the white (OD measured in the absence of serum).

Anti-E_WNV_ neutralizing antibodies are important drivers of WNV protection (8). The production of neutralizing antibodies was evaluated by a WNV microneutralization test. Individuals with neutralizing antibodies (titer>30, defined as the threshold of the WNV microneutralization test) before WNV challenge are shown in **Table 2**. No neutralizing antibodies were observed in the negative control or in the rVHSV-SP_G_E_WNVΔTM_TM_G_ groups, whereas the majority of individuals from positive control groups produced neutralizing antibodies (10/12 and 6/12 for rE_WNV_ and rDIII_WNV,_ respectively). Neutralizing antibody production was also observed in some of the animals vaccinated with rVHSV-SP_G_E_WNV_ and rVHSV-SP_G_E_WNV_TM_G_ (4 and 3 out of 12 mice, respectively). Only one of the 12 mice immunized with the virus rVHSV-SP_G_DIIDIII_WNV_TM_G_ produced neutralizing antibodies. These results show that rVHSVs expressing the entire E_WNV_ at the virion membrane can efficiently induce neutralizing antibodies in a fraction of immunized mice. A strong heterogeneity is observed in groups immunized with rVHSVs. Some of the mice immunized with rVHSV-SP_G_E_WNV_ and rVHSV-SP_G_E_WNVΔTM_TM_G_ show a high anti-E_WNV_ IgG production with OD values close to those observed in the groups immunized with the recombinant proteins (rE_WNV_ and rDIII_WNV_) while others do not develop a specific IgG response (**Figure 7**).

### Serial immunizations with rVHSV expressing entire E_WNV_ partially protect mice against WNV challenge

In order to evaluate if specific WNV immune responses induced upon rVHSVs vaccination were efficient at ensuring protection against WNV, immunized mice received a lethal dose of WNV, Israël-98 strain, by the intraperitoneal route one month after the last immunization.

Virological protection was first investigated. WNV is known to induce a short viremia in mammalian host, peaking at days 3 and 4 after infection in mice. Upon rapid control of WNV peripheral replication by the innate and acquired immune system, no viremia is observed and protection from WNV neuroinvasive disease can be afforded. We therefore examined viremia in mice immunized with rVHSVs encoding E_WNV_ antigens at day 3 post-infection. In the positive control groups immunized with rE_WNV_ or rDIII_WNV_, 92% of the mice (11 out of 12 mice) had no viremia at day 3 after challenge (**Table 3**), whereas the results obtained in the groups immunized with rVHSV-SP_G_DIIDIII_WNV_TM_G_ or rVHSV-SP_G_DIII_WNV_TM_G_ (data not shown) did not statistically differ from the negative control groups. Furthermore, in groups rVHSV-SP_G_E_WNV,_ rVHSV-SP_G_E_WNVΔTM_TM_G_ and rVHSV-SP_G_E_WNV_TM_G_ (data not shown), viremia was detectable in a limited number of mice at day 7 (2 mice out of 12).

**Table 3:** rVSHV-SP_G_E_WNV_ and rVSHV-SP_G_E_WNVΔTM_TM_G_ partially protect mice against a lethal WNV challenge.

Antigens	Clinical protection		Virological protection	
	Number of animals with weight loss ^a^ <10%	Number of surviving animals ^b^	Absence of Viremia ^c^ 3dpi	Absence of Viremia ^c^ 7dpi
TEN 1X	2/12	3/12	2/12	4/12
rE_WNV_	12/12	12/12	11/12	12/12
rDIII_WNV_	11/12	12/12	11/12	12/12
rVHSV	4/12	4/12	0/12	7/12
rVHSV-SP_G_E_WNV_	8/12	8/12	5/12	10/12
rVHSV-SP_G_E_WNVΔTM_ TM_G_	8/12	8/12	4/12	10/12
rVHSV-SP_G_DIIDIII_WNV_TM_G_	3/12	3/12	1/12	7/12

(a) The percentage of weight loss was calculated everyday post-challenge during 15 days. This percentage is based on the variation of weight observed everyday compared to that measured the day of the WNV challenge.

(b) Number of surviving mice on day 21 post-challenge.

(c) WNV genomic RNA was detected by quantitative RT-PCR in the blood of mice collected on days 3 and 7 post-challenge.

**Figure 8:**
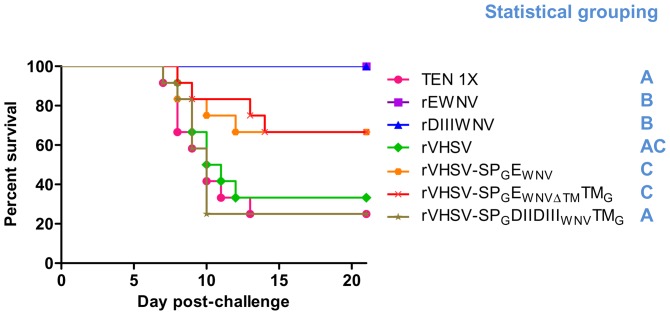
Survival Curves of mice immunized with rVHSV carrying WNV E antigens and infected with a lethal dose of WNV. At 28 days after the last immunization (day 56), immunized mice were challenged intraperitoneally with a lethal dose (1,000 PFU) of WNV (Israël-98 strain). Mice were observed daily for signs of morbidity. For statistical grouping, a comparison of survival between groups was performed with the log rank test on the Kaplan-Meier survival data using GraphPad Prism (GraphPad, San Diego, CA). Groups that were assigned to statistically similar groups (and, thus, share a letter) are not significantly different from each other (P>0.05), whereas those that were not assigned to statistically similar groups are significantly different (P<0.05).

BALB/c mice infected with WNV Israël-98 strain usually suffer from overt clinical disease from day 6 to day 14. A marked weight loss is one the first sign observed and weight loss was considered significant when it exceeded 10% of the total weight of the individual at the day of challenge. No clinical signs (with the exception of one mouse experiencing a weight loss>10%) and a 100% survival rate were observed in the positive control groups (**Figure 8**). On the contrary, in non-protected groups, e.g. in the negative control groups and the groups immunized with rVHSV-SP_G_DIIDIII_WNV_TM_G_, the survival rate was 25% (3 out of 12 mice) (**Table 3** **,** **Figure 8**). Similar result was obtained with rVHSV-SP_G_DIII_WNV_TM_G_ (data not shown) and the empty rVHSV (4 out of 12 mice). A partial clinical protection was conferred upon immunization with rVHSV-SP_G_E_WNV,_ rVHSV-SP_G_E_WNVΔTM_TM_G,_ with survival rates of 66% (8/12). Similar results were recorded when mice were immunized with rVHSV-SP_G_E_WNV_TM_G_ (data not shown).

Serial immunizations with rVHSV-SP_G_E_WNVΔTM_TM_G_ and rVHSV-SP_G_E_WNV_ viruses efficiently protected some of the vaccinated mice against WNV challenge, and clinical protection correlated with an absence of viremia.

## Discussion

The goal of the present study was to generate recombinant VHSV vectors presenting at their surface the E_WNV_ protein or its subdomains and to characterize their immunogenicity and the protection provided after serial subcutaneous inoculations. Upon addition of Novirhabdovirus SP_G_ and TM_G_ up and downstream of the E_WNV_-derived proteins, we successfully recovered five rVSHV vectors incorporating these antigens at their surface. Moreover, we have demonstrated that rVSHV vectors bearing the E_WNV_ protein were capable of inducing Th2-oriented immune responses, affording protection against WNV challenge, in 40 to 50% of the vaccinated mice.

Inserts of up to 1.7 kb, located between the N and P cistrons, were readily added in the VHSV genome by reverse genetics. Corresponding rVSHV vectors displayed the same morphology as parental VSHV and were produced at high titers, with less than 10-fold difference compared to empty rVSHV. Our data on VHSV confirm the flexibility of rhabdovirus genomes that enable the insertion of up to 6 kb of foreign sequence with a high level of expression of the corresponding heterologous protein [34]. The size of rhabdovirus particles containing longer viral genomes were previously shown to increase proportionally [34]. In our study, as the genes of interest were inserted between N and P cistrons it can be postulated that it had a negative effect on the kinetics of viral replication since a gradient of transcription is observed in the Mononegavirales [35].

E_WNV_-derived antigens were efficiently incorporated at the surface of rVHSV particles demonstrating that Novirhabdovirus SP_G_ and TM_G_ sequences were required to target the cell membrane. More precisely, addressing of E_WNV_ at the plasma membrane of rVHSV infected cells was conditioned by the fusion of SP_G_ to the N terminus of E_WNV_. Our results also show that TM_G,_ including the cytoplasmic tail sequence, fused at the C terminus efficiently anchored E_WNV_-derived antigens at the plasma membrane (for rVSHV-SP_G_DIII_WNV_TM_G_ and rVSHV-SP_G_E_WNV_TM_G_). WNV and flaviviruses more generally are known to bud from ER membranes, in which WNV surface proteins, prM and E, are inserted [36], [37]. Because E_WNV_ staining in rVSHV-SP_G_E_WNV_ infected cells was in favour of SP_G_E_WNV_ localizing at internal membranes, we tried to enhance E_WNV_ relocation at the plasma membrane by deleting the E_WNV_ transmembrane region composed of 14 residues (rVSHV-SP_G_E_WNVΔTM_TM_G_) [38]. A deletion of the terminal alpha helix (14 residues) did not improve E_WNV_ anchoring at the plasma membrane, nor its incorporation in the viral particle and staining results in fixed and living cells would rather suggest that the deletion of one of the two alpha helices in E_WNV_ transmembrane region destabilized membrane anchoring of the antigen. It is therefore questionable whether the deletion of the E_WNV_ transmembrane region would promote the surface expression of SP_G_E_WNV_TM_G_ antigen; it would seem more reasonable to delete the stem region as well, this latter region being described to contain ER retention signals for another flavivirus, the serotype 2 Dengue virus [39]. Furthermore, we observed that fusion of E_WNV_ domain II to domain III completely modified DIII-staining pattern and virion incorporation, indicating either the presence of localization signals in domain II or antigen degradation or misfolding associated to domain II-domain III fusion. To our knowledge, this is the first study reporting such a drastic change in domain III localization and antigenicity upon fusion to another protein domain and this aspect would deserve further analysis. Overall, we demonstrated that it was feasible, even if challenging, to incorporate ER-associated proteins to the plasma membrane-budding VHSV vector.

Complete or almost complete E_WNV_ antigens incorporated at the surface of rVHSV vectors proved to be the most immunogenic, eliciting IgG responses in virtually every mice immunized three times with 10 μg total proteins. In contrast, only a subset of mice (0 to 44% of vaccinated mice with rVSHV-SP_G_E_WNV_, rVSHV-SP_G_E_WNV_TM_G_ and rVSHV-SP_G_E_WNVΔTM_TM_G_) developed detectable antibodies that efficiently neutralized WNV in *in vitro* cultures, demonstrating the difficulty at inducing WNV neutralizing antibodies [40]. Surprisingly rVSHV bearing E_WNV_ most immunogenic domains, e.g. domain III or domains II and III, did not elicit any humoral response in vaccinated mice, in contrast to studies reporting antibody secretion after vaccination with recombinant or VLP/virus-associated E_WNV_ domain III [6], [9], [14], [41], [42]. Putative hypothesis could be the misfolding or the inaccessibility of E_WNV_ domains at the surface of rVHSV particles or the injection of insufficient doses. In the current study 10 μg total proteins, with a majority of VHSV structural proteins, have been injected versus 2–100 μg of recombinant domain III. This could explain in part these contrasted results [6], [9], [11], [43]. However it has to be mentioned that the SP_G_E_WNV_TM_G_ antigen, although almost undetectable in Western blot was anyway in sufficient amount to induce detectable antibody responses. Interestingly, one other group also reported poor priming of antibody responses by domain III [44].

As expected with a vaccine platform incapable of replication in mammalians, and therefore resembling chemically or thermally inactivated vaccines, rVSHV vectors mainly induced Th2-oriented immune responses. The clinical protection correlated with an early control of WNV peripheral replication (absence of viremia) and was mainly associated with the induction of neutralizing antibody responses. Such a correlation further emphasizes the importance of priming early effective neutralizing antiviral responses for the control of severe WNV infection as shown by others [31], [45]–[47]. However, it is interesting to note that mice vaccinated with rVSHV-SP_G_E_WNVΔTM_TM_G_ were partially protected against WNV challenge (8 survivors out of 12 animals) in the presence of IgG and absence of detectable neutralizing antibodies (at least in the conditions used in this study), suggesting the induction of poorly neutralizing but protective responses, such as the ones classically obtained against domain II [31], [48].

The present study supports the idea that rVHSV vectors are interesting vaccine platform, eliciting Th2-oriented immune responses and conferring partial protection against a highly virulent WNV strain in most of vaccinated animals. One the most interesting advantage in terms of safety of using Novirhabovirus, like VHSV, as a vaccine platform to vaccinate warm-blood animals is that VHSV is unable to replicate at temperature higher than 20°C and thus there is no requirement to chemically inactivate it before injection in animals. However in contrast the disadvantage of using a non-replicating platform is that several boosts are required to mount an immune and protective response in vaccinated animals. Definitely, presenting E_WNV_ protein at their surface was superior to vaccination with E_WNV_ subdomains and further studies should be designed to enhance E_WNV_ incorporation in rVHSV virions (through the deletion of alpha helices and/or stems in E_WNV_ transmembrane region), to favour proper domain III folding and presentation (through its multimerisation by inserting fused multiple copies of DIII in the rVHSV genome) or to enhance rVHSV immunogenicity through the use of novel adjuvants [49].

## Supporting Information

Figure S1 **Fusion PCR performed to generate the coding sequence of SP_G_DIII_WNV_TM_G._** (A) Two PCR reactions were performed (1 and 2 with the primers SPIHNDIIIF and DIIIVHSTMR, and DIIIVHSTMF and VHSTMR, respectively (Table 1)). (B) An equimolar mixture of the two PCR products was used as template for a third PCR reaction to obtain the final fragment SP_G_DIII_WNV_TM_G_ using the primers SPIHNF and VHSTMR (Table 1). (C) Migration on an 1%-agarose gel of the two intermediary fragments (SP_G_DIII_WNV_ and TM_G_) and the final product (SP_G_DIII_WNV_TM_G_).
(TIF)
