# Supplementary figures and images for: A Recombinant Novirhabdovirus Presenting at the Surface the E Glycoprotein from West Nile Virus (WNV) Is Immunogenic and Provides Partial Protection against Lethal WNV Challenge in BALB/c Mice
Source: PLoS One. 2014 Mar 24;9(3):e91766. doi: 10.1371/journal.pone.0091766 (PMC3963854; doi:10.1371/journal.pone.0091766)

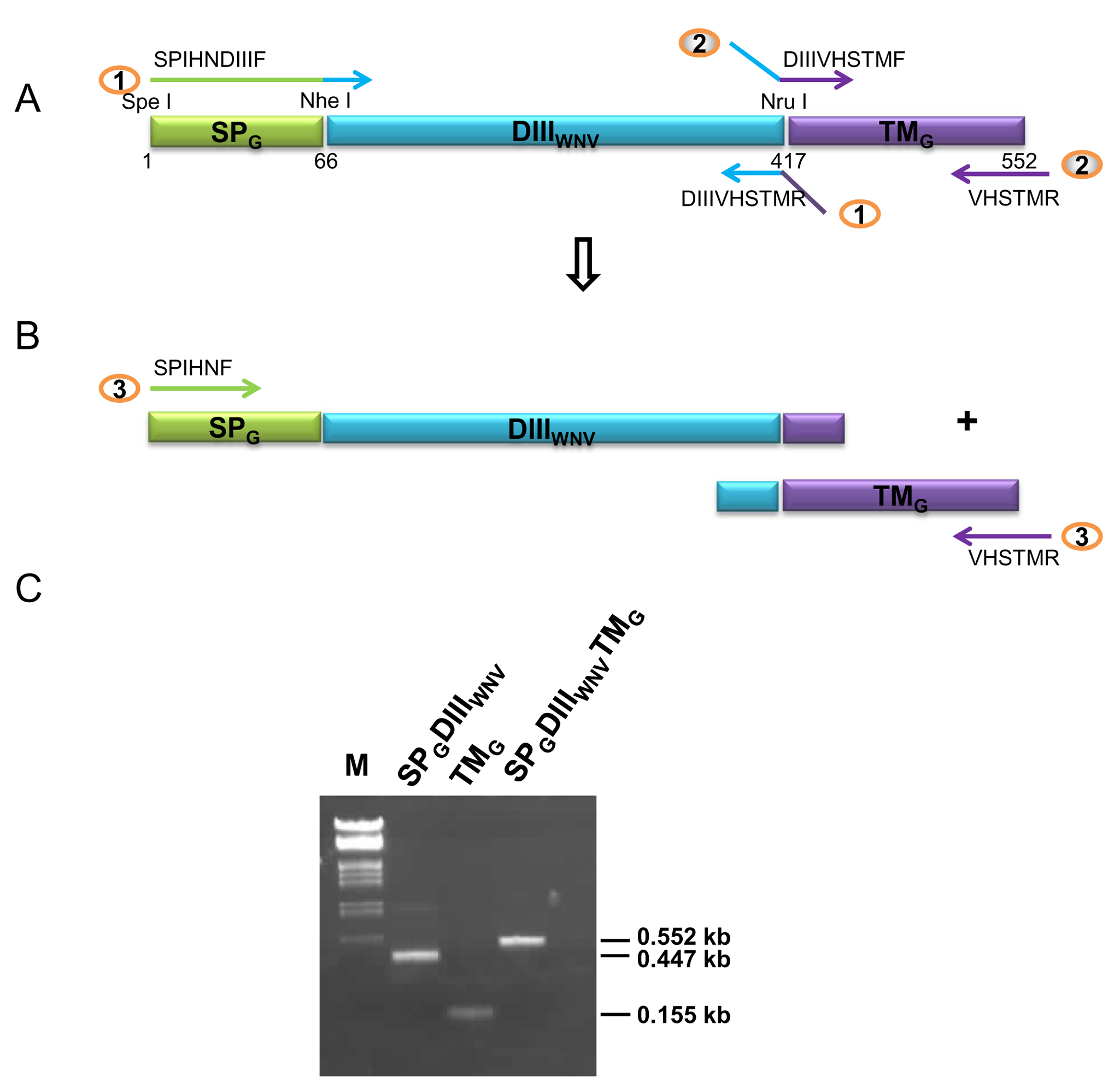

Supplement: Figure S1 — Fusion PCR performed to generate the coding sequence of SPGDIIIWNVTMG. (A) Two PCR reactions were performed (1 and 2 with the primers SPIHNDIIIF and DIIIVHSTMR, and DIIIVHSTMF and VHSTMR, respectively (Table 1)). (B) An equimolar mixture of the two PCR products was used as template for a third PCR reaction to obtain the final fragment SPGDIIIWNVTMG using the primers SPIHNF and VHSTMR (Table 1). (C) Migration on an 1%-agarose gel of the two intermediary fragments (SPGDIIIWNV and TMG) and the final product (SPGDIIIWNVTMG). (TIF) [file pone.0091766.s001.tif]
